# The Structural and the Functional Aspects of Intercellular Communication in iPSC-Cardiomyocytes

**DOI:** 10.3390/ijms23084460

**Published:** 2022-04-18

**Authors:** Eva Kiss, Carolin Fischer, Jan-Mischa Sauter, Jinmeng Sun, Nina D. Ullrich

**Affiliations:** 1 Institute of Anatomy and Cell Biology, Heidelberg University, Im Neuenheimer Feld 307, 69120 Heidelberg, Germany; eva.kiss@uni-heidelberg.de; 2 George Emil Palade University of Medicine, Pharmacy, Science, and Technology of Târgu Mureș, 540139 Târgu Mureș, Romania; 3 Center of Neurology, Department of Neurology and Epileptology, Hertie Institute for Clinical Brain Research, Otfried-Müller-Straße 27, 72076 Tübingen, Germany; carolin.fischer@uni-tuebingen.de; 4 Division of Cardiovascular Physiology, Institute of Physiology and Pathophysiology, Heidelberg University, Im Neuenheimer Feld 307, 69120 Heidelberg, Germany; jan-mischa.sauter@stud.uni-heidelberg.de (J.-M.S.); j.jingle@hotmail.com (J.S.); 5 German Center for Cardiovascular Research (DZHK), Partner Site Heidelberg-Mannheim, 10785 Berlin, Germany

**Keywords:** iPSC-derived cardiomyocytes, gap junctions, connexin 43, intercalated disc, cell replacement therapy

## Abstract

Recent advances in the technology of producing novel cardiomyocytes from induced pluripotent stem cells (iPSC-cardiomyocytes) fuel new hope for future clinical applications. The use of iPSC-cardiomyocytes is particularly promising for the therapy of cardiac diseases such as myocardial infarction, where these cells could replace scar tissue and restore the functionality of the heart. Despite successful cardiogenic differentiation, medical applications of iPSC-cardiomyocytes are currently limited by their pronounced immature structural and functional phenotype. This review focuses on gap junction function in iPSC-cardiomyocytes and portrays our current understanding around the structural and the functional limitations of intercellular coupling and viable cardiac graft formation involving these novel cardiac muscle cells. We further highlight the role of the gap junction protein connexin 43 as a potential target for improving cell–cell communication and electrical signal propagation across cardiac tissue engineered from iPSC-cardiomyocytes. Better insight into the mechanisms that promote functional intercellular coupling is the foundation that will allow the development of novel strategies to combat the immaturity of iPSC-cardiomyocytes and pave the way toward cardiac tissue regeneration.

## 1. Introduction

The technology of induced pluripotent stem cells (iPSC) opened up a new chapter for the field of stem cell research and most importantly for future medical applications. The use of iPSCs is particularly promising in cardiovascular diseases such as myocardial infarction (MI), where iPSC-derived cardiomyocytes could replace scar tissue to restore the functionality of the heart. Recently, major advances have been achieved in this field, providing strong evidence for the high potential of iPSC-cardiomyocytes to regenerate damaged hearts [1]. The use of these cells is still in the pre-clinical stage. A major impediment on the way to their clinical use is that they do not functionally integrate within the host tissue. Besides their differences in shape, size, and different electrophysiological properties, iPSC-cardiomyocytes have a diminished intercellular coupling. This latter feature of iPSC-cardiomyocytes is still insufficiently characterized and the mechanisms behind it are not clearly understood, and both are preconditions for further improvements. The function of the heart is based on contractile activity that requires an adequate propagation of electrical impulses along the conduction pathway. Electric communication between cells is mediated by bursts of action potentials, and gap junctions provide a low resistance pathway for cell-to-cell propagation of the excitation [2,3,4,5]. It is, therefore, reasonable to hypothesize that gap junctions play a central role in the electromechanical incorporation of the transplanted iPSC-cardiomyocytes in the host myocardium. Recent studies have proposed an interesting structural and mechanistic link between the main cardiac gap junction protein connexin 43 (Cx43) and other intercalated disc (ICD) components, suggesting that the structural assembly around gap junctions in the ICD may be of central importance in coordinating the functional coupling of cell excitability and impulse propagation in the working myocardium. This review focuses on gap junction function in iPSC-CMs, and it provides important background information to the structural and the functional limitations of intercellular coupling and viable cardiac graft formation involving the role of Cx43 in these novel cardiac muscle cells.

Our starting point is a ‘precis beyond pros’ of iPSC-cardiomyocytes for cell-therapy, followed by a brief survey of gap junction organization and connexin expression in the normal heart with a short exit to the ICD as the signaling environment of Cx43 [6,7,8]. These will provide the backdrop for explaining the nature of alterations that have been identified in iPSC-cardiomyocytes, giving a résumé about the possibilities to improve Cx43-dependent intercellular coupling abilities.

## 2. Why iPSC-Cardiomyocytes?

Stem cells can be distinguished from other cell types by two important characteristics. First, they can self-replicate. Second and most importantly, they are unspecialized cells that can transform into an array of specialized cells [9,10]. Thus, these cells have the potential to differentiate into cardiomyocytes, to replace dead heart tissue and ultimately to improve the functionality of injured hearts. Both adult stem cells (including, e.g., mesenchymal stem cells or cardiac stem cells) and embryonic stem cells (ESC) have been considered for transplantation into the heart, and they are also currently an important topic of stem cell research [11,12,13]. 

The main argument in favor of using ESCs is their pluripotency. The term pluripotency describes a theoretically unlimited differentiation potential, which gives them an advantage over the multipotent adult stem cell types [14]. However, the clinical use of ESCs is highly debated and it raises ethical issues, since they are generated from cells of a human blastula [15]. Apart from the ethical restrictions on gathering and using human embryonic cells, further problems include possible immune rejection and teratoma formation after implantation [16]. On the other hand, adult stem cells, such as mesenchymal stem cells, are most commonly used in regenerative medicine so far (Zhu 2021). Clinical studies with adult stem cells have shown a good safety profile and an improved cardiac function after their application [17,18]. The fact that they are easily accessible makes them a promising option for cell-based therapies [19]. Moreover, these cells have no risk of teratoma formation [19]. The inability of forming teratomas, however, results from the limited proliferation potency of multipotent cells. This is a problematic point since a large number of cells are needed for cell replacement therapy [20]. The ideal cell type for cell transplantation would consequently need to be available in high yields, while having no risk of teratoma formation, immune rejection, or ethical concerns [21]. So far, there is no cell line that meets all these criteria. However, the new technology of producing human iPSCs (hiPSC) established in 2007 [22] may override these ethical issues and even the risk of immune rejection. Generation of iPSCs starts with collecting somatic cells from patients, such as fibroblasts, through skin biopsies. These cells are reprogrammed into iPSCs by a now standardized protocol typically using viral vectors carrying the key reprogramming factors OCT3, SOX2, KLF4, and cMYC [22]. Through various protocols, iPSCs (similarly to ESCs) are then differentiated into a particular cell lineage. The differentiation holds the benefit that the tumorigenic potential observed by the transplantation of native, undifferentiated stem cells can be largely avoided, thus improving the therapeutic potential of these cells. Even though many differentiation protocols have been developed over the years [23,24,25], the most commonly used technique for creating iPSC-cardiomyocytes is the selective induction or inhibition of gene expression thereby mimicking the effects of signaling pathways that embryonic cells would experience when differentiating into mesodermal tissues such as myocardium. More specifically, this means selective modulation of the Activin/Nodal and the bone morphogenic protein-4 (BMP4) [26] or transforming growth factor-β (TGF-β) and Wnt pathways [27]. This novel technology enables the generation of patient-specific cardiomyocytes in relatively large quantities, altogether representing promising features of iPSC-cardiomyocytes for personalized treatment or modeling of cardiovascular diseases. It allows for the study of developmental biological questions of cardiomyocyte differentiation or for the testing of a drug’s cardiotoxicity, thereby overcoming species–specific differences present in animal models. For example, since iPSCs generated from individuals with genetic disorders maintain the specific anomalies [26], such iPSC-cardiomyocytes are capable of directly modeling inherited cardiac arrhythmias to study the molecular basis of these pathologies, to identify and to assess novel therapeutic strategies for arrhythmia syndromes. Additionally, iPSC-derived cell types can be genetically modified, e.g., through lentiviral transfection, which is useful for studying gain- or loss-of-function in cell models of diseases or for testing a gene therapy approach in vitro [28]. 

All of this proves that iPSCs have made a significant contribution in solving basic research questions. However, implementation of these cells in a clinical context requires significantly higher quality, and above all, safety standards, which usually comes along with higher costs. Besides the concern of structural and functional limitations of intercellular coupling of iPSC-derived cardiomyocytes, which will be discussed in more detail in this review, genomic or epigenomic abnormalities may compromise the differentiation potential, but they may also cause tumorigenesis [29]. Genomic alterations, for instance, can arise during the reprogramming or even during the prolonged culture process [30]. This is a major issue for downstream applications of these cells. Genetic instability or the use of not fully differentiated cells may even lead to an immune response in an autologous transplantation setting, although it is theoretically considered to be safe from an immunological point of view [31,32,33]. Given the high cost and the effort that would thus be required for a safe autologous transplantation, allogeneic transplantation of well-qualified HLA-matched iPSCs from a biobank is more feasible to meet clinical grade conditions [34,35].

In the end, mesenchymal stem cells as well as iPSCs are tempting to use in cell-based therapies of the diseased heart [36,37]. However, the biggest advantage of iPSCs over mesenchymal stem cells is at the same time their biggest disadvantage: the unlimited proliferation capacity and the potential to differentiate into all three germ layers bear the risk of teratoma formation. Nevertheless, the transplantation of pure pre-differentiated iPSCs is a safe method, whereby the advantages of iPSCs ultimately outweigh the disadvantages. In addition, it is worth mentioning that biobanks of pluripotent stem cells could not only be applied for cardiac regeneration but are also applicable on a broad scale for other common diseases associated with derivatives of other germ layers. The ultimate goal of providing novel cardiomyocytes for cell-based cardiac regeneration can only be achieved by eliminating the aforementioned concerns of immune rejection and tumorigenesis, but also having a standardized procedure for the efficient induction of cardiomyocytes lineages from pluripotent stem cells [38].

## 3. iPSC-Cardiomyocytes for Cell Replacement Therapy in the Diseased Post-MI Heart

Prolonged ischemia in the ventricular wall resulting from occlusion of a coronary artery, as is the case in MI, causes massive death of cardiomyocytes. Monocytes infiltrate into the ventricular wall to eliminate dead cardiomyocytes, but also to initiate a repair response based on the recruitment and the activation of resident cardiac fibroblast progenitors at the damaged site [39,40]. These activated fibroblasts, aka myofibroblasts, synthesize large amounts of collagen and other extracellular matrix proteins, forming non-contractile fibrous tissue that progressively transforms into a poorly cellularized and stiff scar [41]. All these post-MI phenomena referred to as ventricular remodeling can lead to detrimental functional consequences from life threatening arrhythmias to end stage heart failure. Ventricular arrhythmias arising from MI-induced electrical remodeling processes remain a leading cause of morbidity and mortality in industrialized countries, even leading to the syndrome of sudden cardiac death [42,43,44]. Studies of post-infarct patients have shown that these arrhythmias generally originate in the border zone of the fibrotic scar connecting to surviving myocardium. They are thought to be associated with the phenomenon of anisotropic reentry, leading to slowed impulse propagation or even localized conduction block caused by fibrotic patches in the diseased myocardium. The progressive electrical remodeling and loss of working myocardium after MI disrupt the coordinated activation and repolarization of the heart, which remains a major challenge for health beyond the possibilities of current therapeutic approaches [45,46]. Current pharmacological and surgical therapies such as primary angioplasty, the introduction of a stent or creation of a coronary artery bypass graft can eventually slow down maladaptive cardiac remodeling, but it cannot revitalize or replace the damaged cardiomyocytes. Thus, an efficient cell-based therapeutic approach involving iPSC-cardiomyocytes to substitute dead or damaged cardiomyocytes of the heart represents a novel but also urgently needed option for these patients. 

A growing number of studies have explored the possibility of transplanting iPSC-cardiomyocytes or mesenchymal stem cells either as single cells or cell sheets to repair the post-MI heart in different animal models [36,37,47]. The most important benefit of iPSC-CMs lies in the control over the differentiation process. While the differentiation of MSCs towards a cardiac phenotype post transplantation is still debated, a possible paracrine effect of MSCs may better explain the majority of improvement in cardiac function after implantation. In contrast, iPSCs can be fully differentiated into cardiomyocytes in a controlled environment in vitro. At the point of transplantation, although still immature, most integral properties such as cardiac ion channels, the existence of a contractile apparatus and cell adhesion proteins as well as electrical signal propagation through gap junctions can be observed, offering the possibility of functional integration into the host tissue. This benefit goes hand in hand with their high availability, as iPSCs can be derived from fibroblasts retrieved from easily accessible skin biopsies of patients. This makes them a prime example of personalized medicine as they are patient-specific, but it also removes some ethical concerns because there is no need for human embryonic material as in the case of hESCs. Most of the studies using iPSC-cardiomyocytes for cardiac implantation after MI in mice, rats, guinea pigs, sheep, or pigs [1,48,49,50] showed a significant decrease in cardiac fibrosis and an improvement in cardiac function following MI. Promising transplantations of iPSC-cardiomyocytes have been performed also on non-human primate hearts. In 2016, Shiba et al. [51] reported the application of allogenic iPSC-cardiomyocytes to repair post MI lesions in the cynomolgus monkey (*Macaca fascicularis*) sharing the identical major histocompatibility complex (MHC) to that of humans. The grafted iPSC-cardiomyocytes were able to electrically couple to the host myocardium and improved cardiac contractile function for at least 12 weeks. Despite these encouraging results, all transplanted animals presented episodes of ventricular tachyarrhythmias [51] as was earlier the case by the transplantation of hESC-cardiomyocytes into post-MI hearts of non-human primates [52,53]. In the latter work, Liu et al. investigated the possible mechanisms behind these arrhythmias in the monkey MI-model. They found that neither high-frequency overdrive nor electrical cardioversion could correct these arrhythmias suggesting the mechanism of re-entry as origin [53]. A later work by Kashiyama et al., compared the effects of MHC-matched and mismatched allogeneic iPSC-cardiomyocyte sheets in the same primate model and concluded that cardiac function can indeed be improved and preserved for up to six months after transplantation. However, in immunohistological stainings of heart sections, GFP-positive cell grafts were detected within the cardiac tissue only up until three months but beyond this time frame after transplantation [54]. 

Together, these results demonstrate that iPSC-cardiomyocytes offer functional benefits to MI-injured hearts in vivo, including in non-human primates. Nonetheless, they also indicate that despite a number of approaches developed to enhance iPSC-cardiomyocytes maturity [55,56], in the current form, iPSC-cardiomyocytes have pro-arrhythmic properties and in general a limited engraftment potential [57,58]. This denotes the absence of adequate electrical incorporation of the grafted cells into the host myocardium. One possible explanation for this observation may lie in the reported insufficiency of iPSC-cardiomyocytes to form electrical junctions with neighboring cells, leading to an inadequate intercellular coupling efficiency. This, in turn, is the consequence of a shortage in gap junctions compared to native cardiomyocytes [59,60]. To override these limitations, iPSC-cardiomyocytes need further optimization in a way that they form an efficient functional syncytium with each other and with native ventricular cardiomyocytes. To better understand the processes that control gap junction formation in iPSC-cardiomyocytes, additional in-depth characterization of their true cardiogenic potential is required in terms of cell excitability and intercellular coupling at single cell level and in multicellular preparations. Comprehensive understanding of the regulatory pathways underlying gap junction formation and modulation in the developing and adult myocardium may open new targets for pharmacological interventions in situations of life-threatening arrhythmias.

## 4. Gap Junctions and Electrical Signal Propagation

Due to the sequential contractile activity of the atria and ventricles, the heart requires a fast and a well-coordinated propagation of electrical impulses. Gap junctions represent efficient low resistance pathways that allow the electrical impulse to flow rapidly and repeatedly between cardiomyocytes, ensuring that their mechanical responses are spatially and temporally synchronized in the healthy heart [61]. Gap junctions are indispensable for the communication between two adjacent cardiomyocytes and especially working ventricular cardiomyocytes are abundantly interconnected by them. Gap junctions are plaques of channels in the sarcolemma, which connect the cytoplasm of two cells and enable the diffusion of ions (K^+^ and Ca^2+^) [62], small molecules (<1 kDa), including those that regulate differentiation, tissue patterning, and development [63,64], and even micro-RNA between adjacent cells [65]. Structurally, two hemi-channels, also called connexons, one per cell, dock together at contacts between cells. Connexons, in turn, are built of six connexin units. The gene family of connexins comprises 21 members in the human genome, and it is highly variable [66]. In general, all connexins have a highly conserved structure including four membrane-spanning segments, two extracellular loops and one cytoplasmic loop with both the N- and the C-terminal regions located in the cytosolic face of the membrane [67,68]. However, the intracellular loop as well as the C-tail region vary in length and sequence in different connexins. Actually, the C-terminal region plays an important regulatory role for the modulation of gap junctions. It is the most variable part of the protein, and it is responsible for the different eponymous molecular weights of connexins. These differences in connexin subunits lead to channels with different regulatory and permeability properties [69], consistent with certain connexin isoforms exclusively expressed in certain organs or tissues. The three major connexin isoforms of the heart tissue are connexin 43 (Cx43), connexin 45 (Cx45) and connexin 40 (Cx40) [70]. They are expressed in characteristic combinations and relative quantities in a chamber-related manner [71]. In addition, their expression may sometimes be cell-specific, so that Cx43 is the only connexin known to be expressed in adult ventricular cardiomyocytes as shown by immunohistochemistry analysis [72,73]. The heterogenous distribution pattern of different connexins of course has a functional significance. Cx40, which generates channels with high conductance, can be found in fast-conducting tissues of the His-Purkinje system, whereas Cx45 generating channels with low conductance are expressed in the atrioventricular node and His bundles [70]. This compartmentalized expression pattern of connexins enables the well-tuned and the sequential spreading of electrical activation from the atria to the ventricular chambers [62].

Gap junctions are highly dynamic structures, channels from the center of a plaque being internalized into vesicular structures called ‘‘annular junctions’’ and probably degraded in lysosomes or proteasomes, while new connexons are added to the periphery of a plaque [74,75]. Correspondingly, gap junction proteins have a rapid turnover rate (half-lives of about 1.5 to 5 h both in vitro and in vivo) [76,77,78], suggesting well-coordinated and regulated synthesis and trafficking of connexin proteins. 

Connexins are translated in the endoplasmic reticulum and progressively oligomerize into a hemi-channel or connexon. This process ends in the trans-Golgi network [79,80,81]. Packed in vesicles, connexons are then transported to the cell surface via microtubules and with the help of the actin cytoskeleton. At the membrane, the vesicles fuse with the plasma membrane [82,83]. Connexons can either remain single non-junctional channels in free membrane areas or they diffuse freely to regions of cell-to-cell contact to find a partner connexon from a neighboring cell and to complete the formation of intercellular channels [84]. Intercellular channels then cluster into gap junction plaques [75].

## 5. Intercalated Discs 

In mature cardiac tissue, gap junctions are preferentially localized at the long end of the cells, as first indicated in 1967 by Revel and Karnovsky [85]. These sites are called intercalated discs and they represent the site of tight and intensive mechanical contact between the ends of rod-shaped cardiomyocytes, where also myofibrils are anchored. There are three types of cell–cell contacts at the intercalated discs: (1) The nexus or gap junctions consisting of Cx43 in ventricular cardiomyocytes allow ion transmission and function as electrical junctions and intercellular tunnel system [72,86]. The other two contacts constitute rather mechanical junctions between cardiomyocytes. (2) The fascia adherens or adherens junctions anchor the actin-containing thin filaments of the myofibrils to the sarcolemma [61]. They are composed of N-cadherin as a transmembrane protein that provides the link to the adjacent cell and a cytoplasmic plaque that contains a set of catenins (i.e., α-catenin, β-catenin, and plakoglobin/γ-catenin) and catenin-related proteins (vinculin, α-actinin, p120cas, ARVCF, and p0071). Thus, they connect the junction to the actin cytoskeleton; and (3) The macula adherens or desmosomes consist of transmembrane spanning desmosomal cadherins (desmoglein and desmocollin), which link the intermediate filaments (desmin) of the cytoskeleton of two adjacent cardiomyocytes through interaction with the cytoplasmic plaque proteins desmoplakin, plakophilin, and plakoglobin etc. [87]. The desmin surrounds and interlinks the Z-discs and connects the contractile apparatus to the sarcolemma. Mechanically spoken, the intercalated discs are found at the ends of myofibrils and translate the sarcomere shortening into cellular contraction. Moreover, they transmit the mechanical force, and they perform the electrical coupling, allowing individual cardiomyocytes to work as syncytium. 

Interestingly, there is increasing evidence supporting that the intercalated disc is, structurally and functionally, not a simple aggregate of independent, separate complexes but the site of a large network of interacting proteins and, therefore, a sensitive area for pathophysiological remodeling [88,89,90,91].

## 6. Gap Junctions in iPSC-Cardiomyocytes

**Figure 1:**
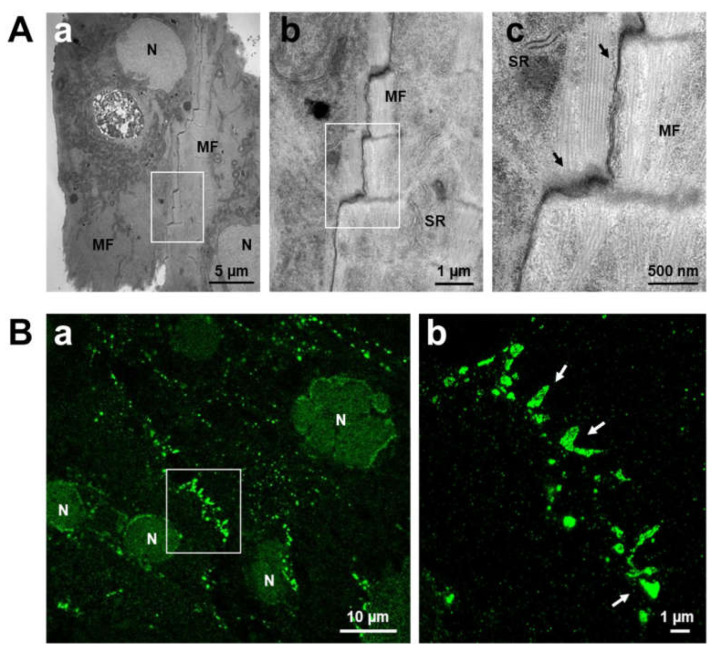
Intercellular connections and intercalated discs in iPSC-cardiomyocytes. (**A**) Ultrastructure of two neighboring iPSC-cardiomyocytes showing the intercalated disc-like structures between both cells reminiscent of a stair case. (**a**) Transmission electron micrograph; (**b**) enlargement of the white box indicated in (**a**), (**c**) detail of (**b**), visualizing points of cell adhesion and gap junction plaques between both cardiomyocyte membranes (arrows). (**B**) Immunolabeling of Cx43 in iPSC-cardiomyocytes. (**a**) Overview image taken by a confocal microscope; (**b**) super-resolution confocal image of the magnified area indicated in (**a**), recorded with a STED microscope (with courtesy to Abberior Instruments GmbH, Heidelberg, Germany). White arrows indicate examples of the appearance of gap junction plaques built from Cx43 proteins. Abbreviations: MF: myofibrils, N: nucleus, SR: sarcoplasmic reticulum.

In iPSC-cardiomyocytes, the role of gap junctions and the intercalated discs are less well understood [92,93]. Since one of the key features of iPSC-cardiomyocytes is their widely described immaturity, they present a rather underdeveloped microarchitecture, lacking typical structures such as transverse (t-) tubules and a clear orientation along a longitudinal axis [94]. Accordingly, the anchor points for myofibril attachment via actin are not limited to specific areas of the sarcolemma, known as intercalated discs, but rather diffusely spread across the cell membrane. As a consequence, there are many areas considered as intercalated disc-like membrane stretches, where neighbouring cells intertwine (Figure 1A(a–c)). These areas are also accompanied by gap junctions (Figure 1A(c)). But in total, the abundance of such intercalated disc-like structures and gap junctions is rather low and unorganized. At the protein level, gap junctions in ventricular-like iPSC-cardiomyocytes consist mainly of Cx43. In the past, the mRNA expression levels of different connexin isoforms were investigated and compared to neonatal connexin expression [59]. These experiments revealed a very low expression of Cx43 in both ESC- and iPSC-cardiomyocytes, which does not mean that these novel cardiomyocytes were of a different ventricular cell type, since other cardiac connexins, such as Cx40, Cx45 and Cx30.2, were even less abundant in these cells. Despite the low amount of Cx43 in iPSC-cardiomyocytes, immunocytochemical analysis revealed sarcolemmal expression (Figure 1B(a)) and plaque formation (Figure 1B(b)) indicative of the organization of gap junctions. The function of gap junctions was also confirmed in dye coupling experiments demonstrating that iPSC-cardiomyocytes form a functional syncytium and couple to neighbouring cells via intact gap junctions [59,60,95]. However, according to the rather low Cx43 expression in comparison with neonatal cardiomyocytes, intercellular coupling is significantly reduced in these novel cardiomyocytes, leading to slow electrical signal propagation [95]. The statement on weak intercellular coupling is based on functional experiments such as dye coupling for metabolic exchange and recordings of multi-electrode arrays (MEAs) for the assessment of electric coupling, respectively, which our group has published previously [60,95]. In these experiments, we demonstrated that intercellular coupling via gap junctions in iPSC-CMs is very low, especially when compared to neonatal cardiomyocytes, a comparable cardiac cell model in an early developmental stage of maturation. This is also in accordance with immunofluorescence images showing low expression levels of Cx43 in iPSC-CMs. Electrophysiological recordings confirmed the slow electrical signal propagation with conduction velocities of about 5 cm/s in stem cell-derived vs. 35 cm/s in primary neonatal cardiomyocytes [95]. Moreover, the unspecific distribution pattern of the sarcolemmal Cx43 expression favours diffuse signal spreading across a monolayer of cardiomyocytes, thereby circumventing cell areas of low conductance. Translated into a more clinically relevant context, this form of electrical signal spreading suggests a high proarrhythmogenic behaviour of these cells [51]. Indeed, several in vivo experiments using cardiomyocytes derived from stem cells (ESCs and iPSCs) for implantation into healthy control and MI-hearts demonstrated that these grafted cells promoted the local development of irregular electrical signals [51,52,53,96,97,98]. Despite impressive improvement in cardiac function of infarcted hearts from macaques especially at the level of ventricular function and ejection fraction, graft-induced arrhythmias were prevalent [53]. The association of ventricular arrhythmias with the grafted cells suggests that functional integration of these cells in the host heart may be rather insufficient, leading to reduced intercellular coupling. These data are in strong accordance with the findings that in controlled in vitro cell experiments, the conduction velocity of electrical signal propagation is particularly slow in pure stem cell-derived cardiomyocytes cultures as well as in mixed cultures with primary cardiomyocytes [95]. Moreover, a study by Roell et al., demonstrated a strong dependence of the incidence of ventricular tachyarrhythmias on the expression of Cx43 and, thus, on the formation of functional gap junctions [99]. These data support the idea that intercellular coupling between newly engineered cardiomyocytes needs to be improved to ensure safe electrical signal propagation across the engrafted heart.

## 7. Improvement of Gap Junction Formation and Intercellular Coupling in iPSC-CMs

Successful and stable implantation of iPSC-cardiomyocytes is strongly limited by the currently weak coupling properties not only between iPSC-cardiomyocytes themselves, but also in connection with native cardiac tissue. Therefore, it is of great interest and importance to enhance Cx43 expression and gap junction formation on the one hand, but also to try and achieve a more specific membrane expression pattern of them in iPSC-CMs on the other hand in order to establish a defined route of signal propagation. The difficulty of this purpose is of course related to the general lack of a mature microarchitecture and long axis formation of these cells when grown on planar cell culture dishes. Therefore, many attempts have been undertaken to evoke a longitudinal orientation in iPSC-CMs. In multicellular preparations, great results have been achieved by the now famous technology of engineered heart tissues preparations [100,101]. In this and similar experimental settings, iPSC-cardiomyocytes are grown into long muscle stripes, connected to a stretching device, and rhythmically paced by electrical stimulation [101,102,103]. Despite significant maturation processes that iPSC-cardiomyocytes experience under these conditions, including long axis formation within the novel functional syncytium, a specific orientation of Cx43 expression towards the end poles of the now elongated cells has not yet been observed. The fact that directed Cx43 expression towards the short edges does not happen, even though a clear cell axis has developed during structural remodeling and cell elongation, is rather surprising. If we consider that in adult ventricular cardiomyocytes, Cx43 and thus gap junctions locate near adherens junctions and actin attachment points at the intercalated discs, one would expect that a concentration of the myofibril-membrane attachment towards the cell poles will trigger gap junction clustering in this vicinity as well. However, the exact mechanisms underlying the specific delivery of Cx43 and the localization of gap junctions at intercalated discs still remain largely elusive. This observation clearly tells us that gap junction formation and localization are truly complex mechanisms that we are only slowly starting to discover. Especially one phenomenon, namely the lateralization of Cx43 in diseased myocardium, leading to conduction deficiencies and the development of arrhythmias, is not yet understood [89,104,105]. It will be interesting to test whether novel iPSC-cardiomyocytes may serve as an experimental cardiac cell model to investigate the control and modulation of sarcolemmal Cx43 expression. 

In a recent publication, we have reported the structural and functional effects of enhancing Cx43 expression in iPSC-cardiomyocytes [60]. Forced Cx43 expression leads indeed to increased sarcolemmal localization and augmented functional gap junction formation. Under these experimental conditions, cells were not only better coupled with each other, but also showed increased excitability, which can be explained by larger Na^+^ currents of the voltage-gated Na^+^ channel (Na_v_1.5). Interestingly, it has already been postulated previously that enhanced Cx43 membrane expression triggers enhanced Na_v_1.5 trafficking [88,106,107], suggesting a functional link between two important processes in electrical signal propagation: high cell membrane excitability by voltage-gated Na^+^ channels and optimal intercellular coupling via low-resistance channels, the gap junctions [60,83,106,107]. As a consequence, conduction velocity was significantly faster and the electro-mechanical activity was spatially and temporally highly synchronized in iPSC-cardiomyocytes. It will be interesting to further elucidate gap junction function in iPSC-cardiomyocytes. In light of the fast turnover rate of Cx43 proteins, post-translational modifications such as the phosphorylation state regulated by the activity of different kinases or phosphatases may efficiently control connexon assembly, function, and internalization, depending on the actual needs and the metabolic state of the cells [67,78,108,109]. However, since these processes are rather short-term adaptations to the physiological conditions, they might not persistently affect gap junction formation. For long-lasting effects on Cx43 expression control, one might expect modifications at the transcriptional level or even beyond. Previous reports suggest that these young iPSC-cardiomyocytes are under tight control of micro-RNAs (miRNA), possibly a general feature of developing cells. One possibility to permanently enhance gap junction formation may be to reduce the activity or expression of miRNAs that control Cx43 mRNA translation, such as for example the miR-1/206 or the miR133 families of miRNAs [110,111]. However, at the current state we can only speculate about the many mechanisms controlling or limiting Cx43 expression in iPSC-cardiomyocytes. 

## 8. Conclusions

It goes without saying that targeted modulation of gap junction formation and function will represent a powerful tool to improve the therapeutic potential of iPSC-cardiomyocytes for their anticipated role in cardiac cell replacement therapies. Apart from the required connection with native host cardiomyocytes, these cells will also have to be robust enough to withstand a hostile environment in the diseased heart, possibly presenting different degrees of tissue inflammation, starting development of fibrotic patches, apoptotic cells, and activated cytokines. The reaction of iPSC-cardiomyocytes to such a challenging microenvironment, and specifically the establishment and the behaviour of intercellular junctions will have to be evaluated under these conditions as well. Therefore, apart from the large gain of knowledge, a better understanding of the subcellular processes regulating gap junction formation in iPSC-cardiomyocytes may not only help to identify new targets for pharmacological interventions to reduce the burden of arrhythmias in diseased hearts. Moreover, enhanced gap junction formation will also add to the functional maturation of iPSC-cardiomyocytes and enhance their potential for successful functional integration into host cardiac tissue after implantation. 

## Data Availability

Not applicable.
